# Different Polyubiquitinated Bodies in Human Dendritic Cells: IL-4 Causes PaCS During Differentiation while LPS or IFNα Induces DALIS During Maturation

**DOI:** 10.1038/s41598-017-02090-8

**Published:** 2017-05-12

**Authors:** Daniela Montagna, Patrizia Sommi, Vittorio Necchi, Agostina Vitali, Enrica Montini, Ilaria Turin, Daniela Ferraro, Vittorio Ricci, Enrico Solcia

**Affiliations:** 1 Laboratory of Immunology Transplantation, Foundation IRCCS Policlinico San Matteo, Pavia, 27100 Italy; 2 Department of Clinical-Surgical, Diagnostic and Pediatric Sciences, University of Pavia, Viale Brambilla 74, 27100 Pavia, Italy; 3 Department of Molecular Medicine, Human Physiology Unit, University of Pavia, Via Forlanini 6, 27100 Pavia, Italy; 4 Department of Molecular Medicine, Pathologic Anatomy Unit, University of Pavia, Via Forlanini 16, 27100 Pavia, Italy; 5 Centro Grandi Strumenti, University of Pavia, Via Bassi 21, 27100 Pavia, Italy; 6 Pathologic Anatomy Unit, IRCCS Policlinico San Matteo, Pavia, 27100 Italy

## Abstract

Two types of polyubiquitin-reactive cytoplasmic bodies, particulate cytoplasmic structures (PaCS) and dendritic cell (DC) aggresome-like induced structures (DALIS), were analyzed by electron microscopy, immunocytochemistry, immunoblotting, and flow cytometry in DC obtained from human blood monocytes incubated with GM-CSF plus IL-4 (IL4-DC), GM-CSF plus IFNα (IFN-DC), or GM-CSF alone (GM-DC), with or without LPS maturation. PaCS developed as monomorphic aggregates of proteasome-reactive barrel-like particles only in ribosomes-rich cytoplasmic areas of differentiating IL4-DC. In contrast, DALIS formed as vesicular bodies storing K63-linked ubiquitinated proteins by coalescence of increased endosomal structures, in IFN-DC or after LPS maturation of GM-DC. DALIS-forming cells showed incomplete morphological and functional DC-type differentiation when compared to PaCS-forming IL4-DC. PaCS and DALIS may have different function as well as different origin and cytochemistry. DALIS may be a transient accumulation site of potentially antigenic polyubiquitinated proteins during their processing and presentation. PaCS are found under physiologic or pathologic conditions associated with increased/deranged protein synthesis and increased ubiquitin–proteasome activity. Given its high heat-shock protein content PaCS may work as a quality control structure for newly synthesized, cytosolic proteins. This comparative analysis suggests that PaCS and DALIS have distinctive roles in DC.

## Introduction

Two types of cytoplasmic structures storing polyubiquitinated proteins have been reported in dendritic cells (DC): proteasome-rich particulate cytoplasmic structures (PaCS) and DC aggresome-like induced structures (DALIS). PaCS have been detected in fetal or pathologic tissues due to chronic infection, genetic diseases or a variety of solid, hematopoietic and neuroblastic neoplasms, as well as in some neoplastic cell lines^1–6^. Several PaCS-associated conditions show evidence of overexpression/overfunction of growth factors and/or their receptors. For example, epidermal growth factor receptor overexpression and overactivity in pancreatic serous microcystic adenoma^7^ and in *Helicobacter-pylori*-infected gastric epithelium^8^, or activation of trophic factors in fetal tissue development and differentiation^9, 10^. Several cytokines are commonly used *in vitro* to obtain morphologic and functional differentiation of immunocompetent cells, such as GM-CSF plus IL-4 or IFNα for DC^11–13^, and IL-2 or IL-15 for NK cells, where PaCS have been observed^5^. Thus, the hypothesis that trophic factors and cytokines may have a role in PaCS development was suggested.

Cytoplasmic bodies accumulating polyubiquitinated proteins and p62 protein (sequestosome 1) were first described as DC aggresome-like induced structures (DALIS) by confocal immunofluorescence microscopy after maturation of DC with microbial products, especially LPS^14, 15^. They were subsequently also reported as ALIS in macrophages and several other cell lines, as a consequence of stressful conditions^16, 17^. In professional antigen-presenting cells (APC), DALIS are considered as transient accumulations of potentially antigenic polyubiquitinated proteins *en route* to processing and presentation on the cell membrane as MHC-molecule-bound peptides. In keeping with this interpretation, transmission electron microscopy (TEM) has shown that those structures consist of aggregates of vesicles, mostly endosomal and autophagosomal in origin, that store HLA-related molecules^18^.

We aimed to ascertain whether any structural or cytochemical relationship exists between PaCS and DALIS, and to clarify the role of cytokines and microbial products in their origin and development. We investigated human DC during differentiation and maturation *in vitro* by immunogold TEM, confocal immunofluorescence microscopy, biochemical analysis, and functional markers expression. DC from blood monocyte precursors were treated with GM-CSF with (IL4-DC) or without (GM-DC) addition of IL-4, and with or without subsequent LPS maturation, according to currently established protocols^11, 12^. Addition of IFNα to GM-CSF is highly effective in enhancing DC function, with special reference to viral and tumor antigen cross-presentation^13, 19^, therefore, we also tested GM-CSF plus IFNα (IFN-DC), simultaneously providing DC differentiation and maturation. From our observations, we conclude that PaCS and DALIS are ultrastructurally, cytochemically and functionally different structures. IL-4 exerted a specific role in GM-CSF-treated cells, promoting PaCS induction during DC differentiation, whereas IFNα or LPS favored the formation of DALIS in maturing DC.

## Results

### Phenotype characterization of developing human DC

**Figure 1:**
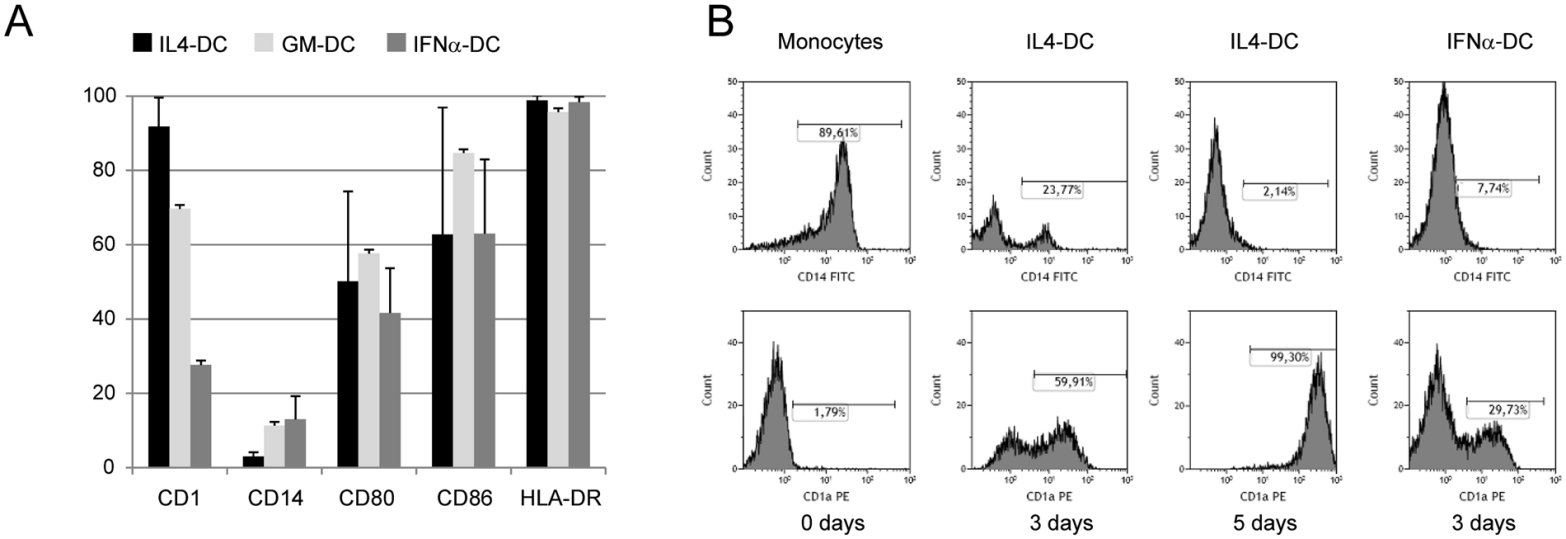
Evaluation of DC phenotype. (**A**) Mean and SD of percentage of surface antigens expressed by DC, generated using different culture conditions. Monocyte-derived DC generated after incubation with GM-CSF and IL-4 (black column) or GM-CSF alone (light grey column) were evaluated after 5-day culture. DC generated after incubation with GM-CSF plus IFNα (dark grey column) were evaluated after 3-day culture. (**B**) Representative flow histograms of CD1a and CD14 expression in fresh monocytes, after 3 and 5 days incubation with GM-CSF plus IL-4, or 3 days with GM-CSF plus IFNα. Percentages of positive cells were evaluated by subtracting values obtained from unmarked cells used as negative controls.

We performed immunophenotypic analysis of CD14, CD1a and of a series of membrane molecules involved in antigen presentation, such as CD80, CD86 and HLA-DR, in monocyte-derived cells. The cells were analyzed fresh and at various times during their DC differentiation, from 7 h until the optimal differentiation time of 5 days for IL4-DC and GM-DC and 3 days for IFN-DC^12, 19^. After 5 days treatment with GM-CSF plus IL-4 (IL4-DC), there was an increase in DC differentiation antigen CD1a in >90% of cells, which was associated with the disappearance of the monocytic marker CD14 (Fig. 1A). Immunophenotypical characterization of IFN-DC after 3 days treatment showed that expression of CD1a (mean 27%, SD 2%) was reduced compared to that in IL4-DC, while a small percentage of CD14 (mean 13%, SD 6%) was retained. Analysis of GM-DC after 5 days treatment showed a mean CD1a percentage intermediate between that of 5-day IL4-DC and 3-day IFN-DC, with persistence of a small percentage of CD14, comparable to that observed in IFN-DC. HLA-DR was highly expressed by almost all cells, irrespective of treatment, while CD80 and CD86 were present in a high percentage of cells, although variable in the case of IL4-DC and IFN-DC. The expression pattern of CD1a and CD14 antigens over differentiation time is shown in Fig. 1B. It appears that DC differentiation is less complete in GM-DC and, especially, in IFN-DC compared to IL4-DC, while no obvious difference emerges from surface expression of molecules directly involved in antigen presentation.

### PaCS induction by GM-CSF plus IL-4

**Figure 2:**
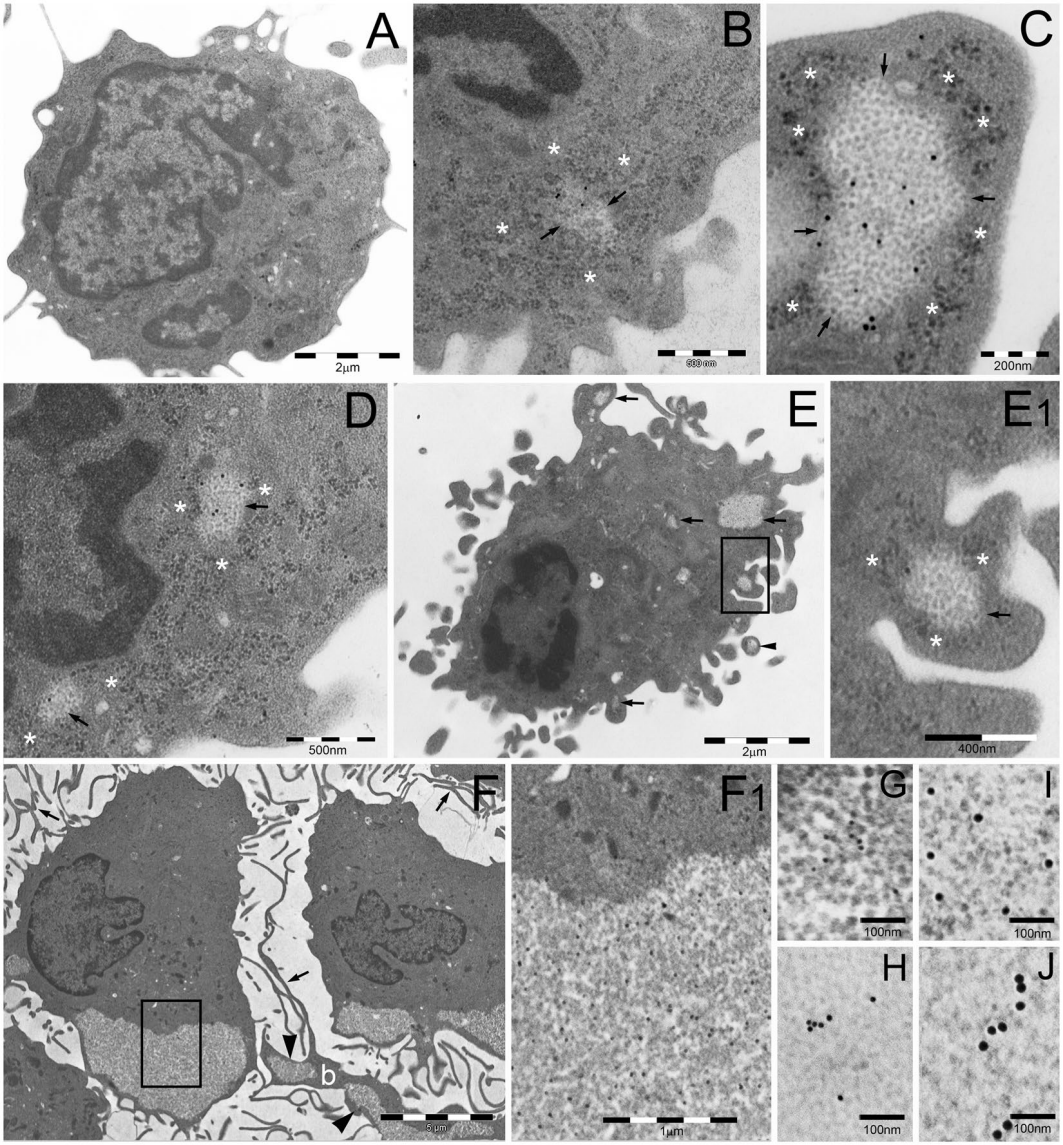
TEM analysis of PaCS development in IL4-DC. (**A**) Untreated human blood monocyte showing moderately developed cytoplasm with only a few organelles and cell protrusions. Cells treated with GM-CSF plus IL-4 for 24 h (**B,C**) or 48 h (**D,E**) showing early PaCS (arrowed borders) development inside ribosome-rich cytoplasmic areas in the absence of ER cisternae. Note FK1-reactive polyubiquitinated proteins (**B,E**) and 20 S (**C,D**) immunogold deposits in PaCS formed by moderately electron dense, barrel-like particles, to be compared with more dense and irregular, PaCS-sorrounding, ribosome particles (asterisks). The cell in (**E**) shows abundant cytoplasm with short hobnail-like protrusions and scattered PaCS (arrows); one of which (boxed) inside a protrusion is enlarged in (E1). Also note in (**E**) a detached PaCS-storing vesicle (arrowhead). Large PaCS are seen in DC after 5 days treatment with GM-CSF plus IL-4 (**F** boxed area enlarged in F1 to show PaCS barrel-like particles with selective 20S immunogold reactivity). Note in (**F**) long and thin cell protrusions (arrows) typical of well-differentiated DC and a long bleb (b) filled with PaCS (arrowheads). 19S proteasome (**G** 10 nm gold particles), HSP40 (**H**, 15 nm), HSP70 (**I**, 20 nm) and HSP90 (**J**, 20 nm) immunoreactivity was found in enlarged portions of PaCS taken from sections adjacent to (**F**).

Investigation of control human blood monocytes by TEM showed the presence of a relatively indistinct cytoplasm with moderately developed organelles (Fig. 2A). At this stage, PaCS were virtually absent. Indeed, only in a very limited number (1%) of cell profiles, we detected few, small (<200 nm maximum diameter) PaCS, accounting for a minimal fraction of the total cytoplasmic area (Table 1).

**Table 1:** PaCS development during GM-CSF+IL4 induced DC differentiation from human blood monocytes.

Treatment (days)	Cell profiles with PaCS (%)	Total cytoplasmic area (μm^2^)	Total PaCS area (μm^2^)	PaCS/cytoplasm (%)
0	1	1923	0.20	0.01
1	5	2414	0.84	0.03
2	31	3015	24.58	0.82
5	82	5638	1095.19	19.43

Data from a representative experiment on 100 randomly selected TEM cellular profiles for each treatment time.

After 7, 14 or 24 h treatment with GM-CSF plus IL-4, no substantial change was observed in terms of cell differentiation. A moderate increase of FK1-reactive polyubiquitinated proteins was seen sparse in the cytoplasm of some cells after 24 h. A slight increase in PaCS frequency (5% of cell profiles), still small in size (100–300 nm in diameter), was seen after 24 h. Importantly, we found that the smallest PaCS, developing after 24 or 48 h treatment, were always localized in close connection with free (poly)ribosomes, in the absence of ER cisternae (Fig. 2B–D). PaCS were characterized by moderately electron-dense barrel-like particles, ~13 nm thick and 15–40 nm long (depending on barrel orientation inside the section), and by a selective immunogold reactivity for 19S and 20S proteasomes, ubiquitin, FK1-positive polyubiquitinated proteins and HSP40, 70 and 90 chaperone molecule antibodies (Fig. 2C–J).

PaCS frequency and size increased substantially after 2 days of treatment (involving about 30% of cell profiles and 200–400 nm in diameter). They progressively reached maximum development after 3–6 days, when PaCS were found in about 80% of cell profiles with a mean diameter of 300–500 nm, occasionally up to 1 μm (Fig. 2F), representing about 20% of the total cytoplasmic area (Table 1).

DC differentiation became evident after 2–3 days, with numerous short, hobnail-shaped cell processes (Fig. 2E). Differentiation reached its maximum at 5–6 days, when the cells showed abundant cytoplasm with many thin mitochondria, moderately developed Golgi and endosomes, abundant free ribosomes, few and short RER cisternae, and numerous, thin, long and curved cell processes (Fig. 2F). At this point, a few PaCS entered cytoplasmic blebs (Fig. 2E,F) and formed membrane vesicles (so called “ectosomes”^20^) filled with polyubiquitinated proteins, proteasomes and chaperone molecules. Occasionally they were found detached from the cell (Fig. 2E).

**Figure 3:**
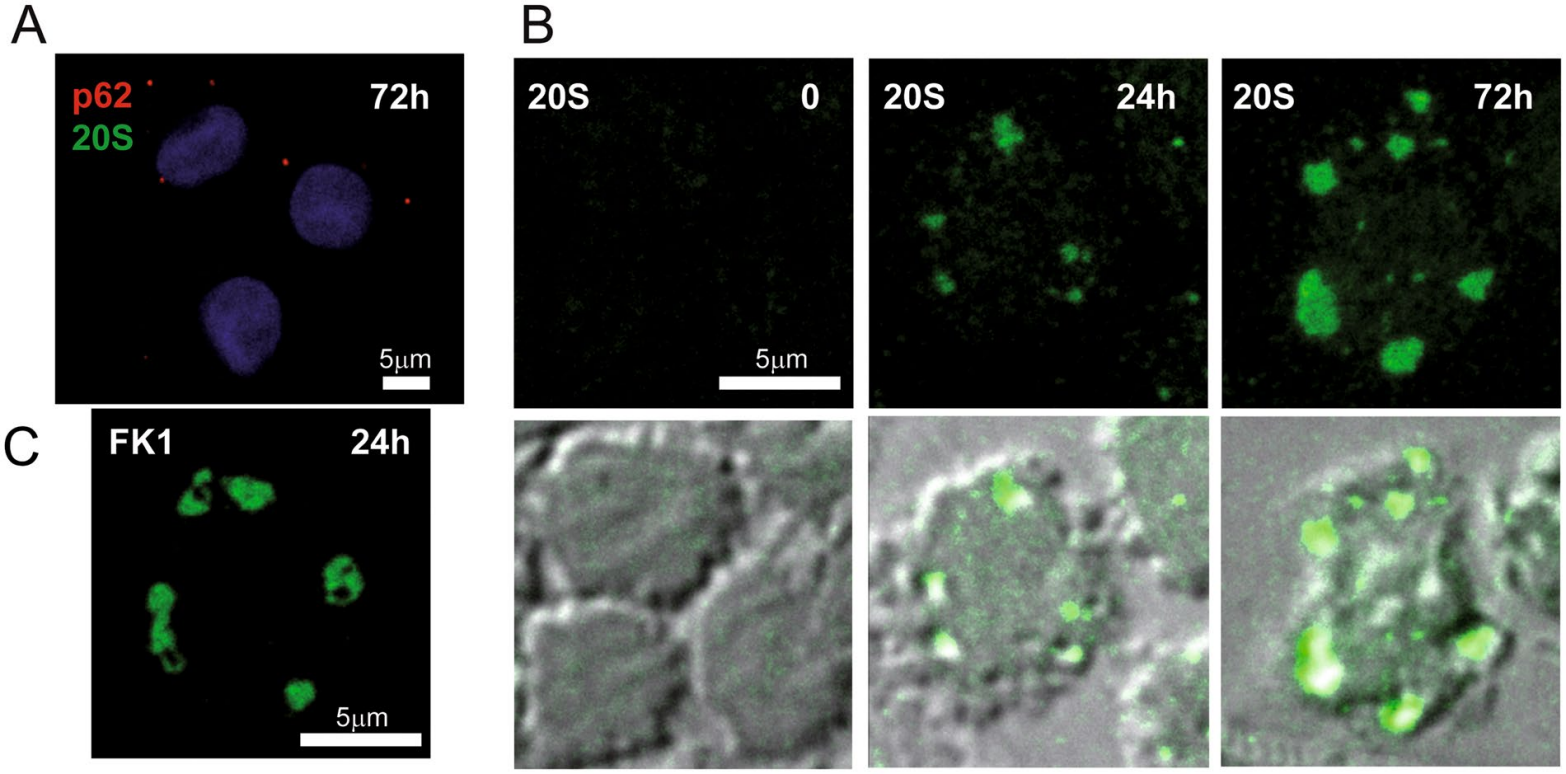
Confocal microscopy of PaCS in IL4-DC. (**A**) IL4-DC incubated for 72 h with GM-CSF plus IL4, processed according to the common sample preparation for confocal microscopy procedure and immunostained with proteasome 20S (green) and p62 (red) antibodies. No positive 20S immunofluorescent structures were visible, while a few small p62-storing fluorescent bodies (red), likely corresponding to autophagosomes, appeared in some cells. (**B**) IL4-DC incubated for 0, 24 or 72 h, fixed, and processed as for TEM and immunostained for 20S proteasome (green). Proteasome-reactive PaCS were visible after 24 and 72 h incubation, but not in control, untreated monocytes. Corresponding phase-contrast images are shown below to help identify the cells. (**C**) Immunofluorescent PaCS were also seen in IL4-DC after 72 h incubation, after immunostaining for polyubiquitinated proteins with FK1 antibody.

By confocal microscopy immunofluorescence, no PaCS-type structures selectively positive for proteasome (19S or 20S antibodies) and polyubiquitinated proteins (FK1 or FK2 antibodies) were detected in cells incubated with GM-CSF plus IL-4 for 3–6 days, and then shortly fixed in formaldehyde and permeabilized according to the standard confocal microscopy procedure. In contrast, 19S, 20S, and FK1-reactive cytoplasmic structures resembling PaCS in size, morphology, cytoplasmic distribution, and development stage were seen by confocal microscopy when semithin (1 μm) resin sections obtained from the same aldehyde/osmium-fixed samples used for TEM investigation were analyzed (Fig. 3). This confirmed the TEM results concerning PaCS. These findings are also in keeping with the previously reported failure of conventionally prepared confocal microscopy specimens to preserve PaCS which, on the opposite, can be preserved and detected after combined aldehyde and osmium treatment, a much stronger fixation^5, 21^.

**Figure 4:**
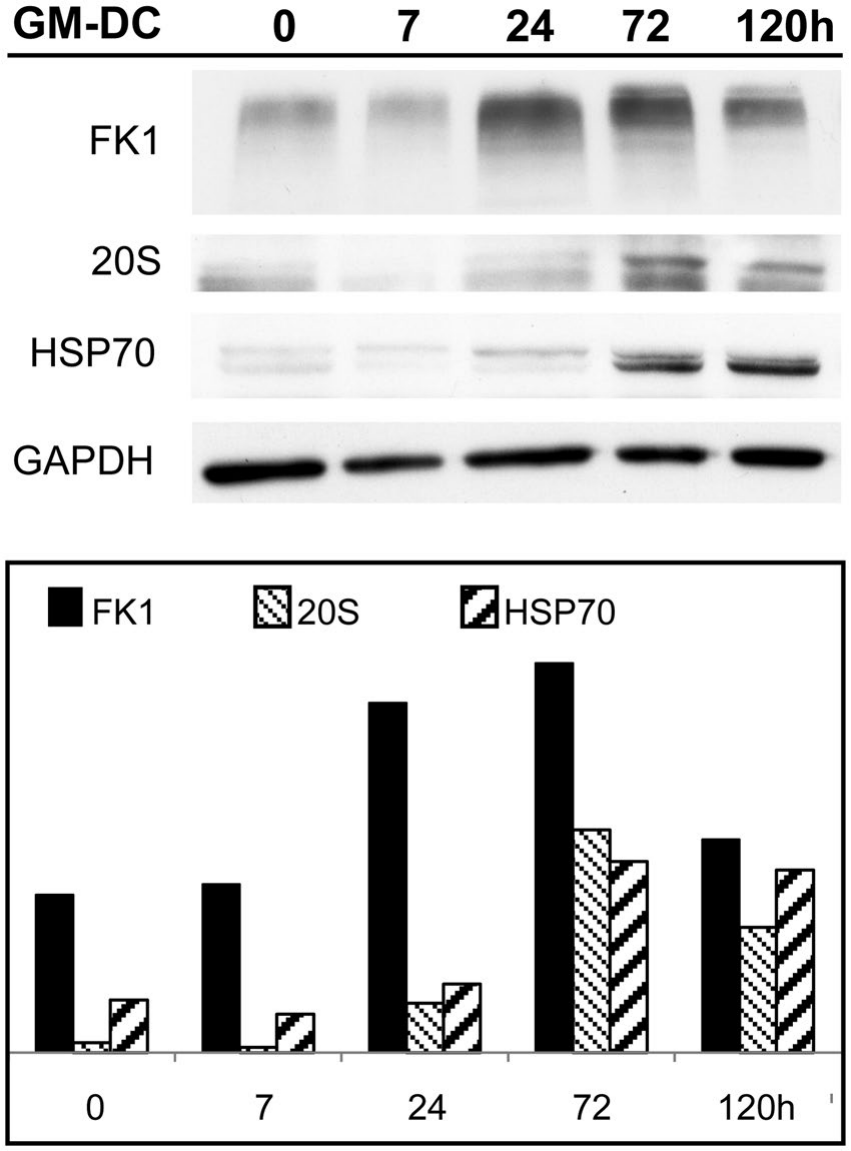
Biochemical evidence of PaCS development. Immunoblotting of DC lysate at various stages of differentiation from their peripheral blood monocyte precursors treated with GM-CSF plus IL-4 for 0–5 days. The histogram shows FK1, 20S and Hsp70 quantitation normalized for protein loading (GAPDH). Note the increment in the levels of FK1-reactive polyubiquitinated proteins and 20S proteasomes starting from 24 h and that of Hsp70 starting from day 3.

PaCS development observed by TEM and confocal microscopy was also confirmed by immunoblotting, for which the levels of PaCS components like FK1, 20S and HSP70 increased over time during DC differentiation (Fig. 4). When extracts obtained from untreated control monocytes or cells previously treated with GM-CSF plus IL-4 from 7 h up to 5 days were analyzed, FK1 and 20S antigens started increasing after 24 h treatment and peaked at 3 days, when HSP70 was also increased.

### PaCS regression in IL4-DC after cytokines withdrawal

**Figure 5:**
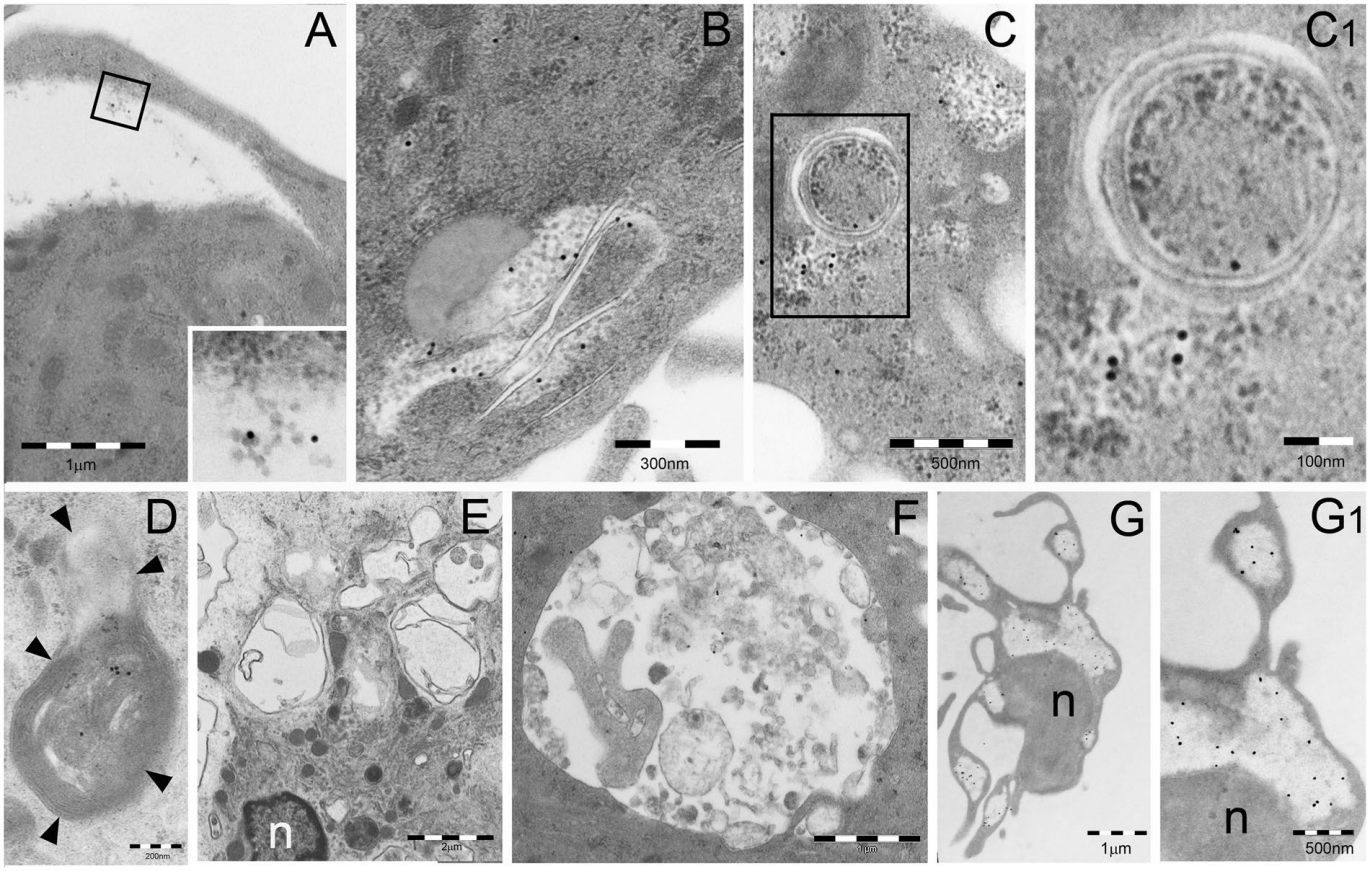
TEM analysis of PaCS autophagy and bleb formation in IL4-DC after IL-4 withdrawal. TEM of cells incubated for 5 days with GM-CSF plus IL-4 and then for 3 (**A–D**) or 5 (**E–G**) days in RPMI–FBS without cytokines. (**A**) A clear area, not delimited by membrane, was seen in the cytoplasm of a DC. This was identified as an empty PaCS, due to its residual barrel-like particles and 20S immunogold reactivity, visible at higher magnification in the inset. (**B–D**) Autophagic double or multilayered membranes penetrate and wrap/envelope cytoplasmic areas containing FK1-positive PaCS. See structural details in the enlargement (C1) and p62 immunoreactivity of an autophagosomal vesicle with fused multilayered membranes in (**D**). (**E**–**G**) A collection of empty autophagic vesicles and no PaCS are seen in (**E**), while in (**F**) a single-membrane vesicle shows heterogeneous, lysosome-type dense bodies and sparse, residual immunogold reactivity for the autophagic marker LC3. (**G**) FK1-reactive PaCS were still recognized in the cytoplasm as well as in the cell blebs, although most barrel-like particles were dissolved. Nuclear and cytoplasmic densification, with loss of details and organelles, were indicative of ongoing apoptosis. See enlargement in (G1). n, nucleus.

In cells incubated for 5–6 days with GM-CSF plus IL-4, no substantial change was found by TEM 24 h after cytokine withdrawal. After 48–72 h, signs of PaCS regression with focal or diffuse loss of barrel-like particles, coupled with loss of immunogold reactivity for proteasome and polyubiquitinated proteins, were observed in several cells (Fig. 5A). In addition, p62 and LC3 reactive autophagosome vesicles appeared in many cells, surrounding and engulfing cytoplasmic areas with residual PaCS within their double or multilayered membranes (Fig. 5B–D). Four to five days after cytokine withdrawal, when apoptosis was extensive, massive autophagy and PaCS loss were seen in surviving cells (Fig. 5E,F). However, in some of the apoptotic cells PaCS were still recognized by their immunocytochemical markers (Fig. 5G).

### DALIS development

DALIS development was seen when GM-DC were further treated with LPS or when IFNα was added to GM-CSF during differentiation, even without further LPS maturation treatment.

#### GM-CSF followed by LPS

**Figure 6:**
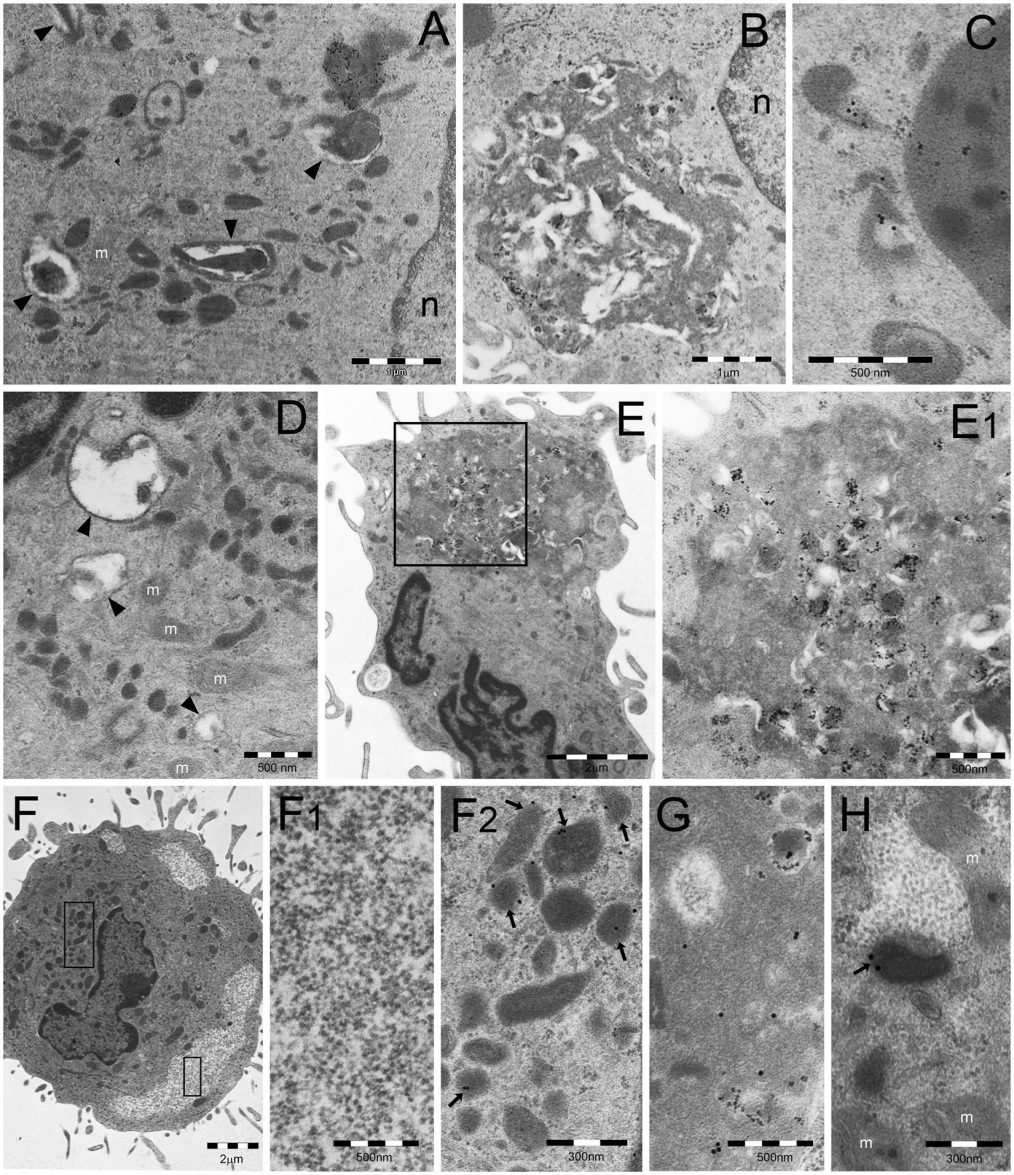
TEM analysis of DALIS development in GM/LPS-DC (**A**–**C**) or IFNα-DC (**D**–**E**). (**A**) Numerous endosomes of various shape and density and autophagic vesicles (arrowheads) are scattered in the cytoplasm of a DC incubated with GM-CSF for 5 days. (**B**) In cells first incubated with GM-CSF as in (**A**) and then treated with LPS, DALIS appear formed by a collection of vesicles with interposition of amorphous material and dense bodies. DALIS likely result from aggregation of autophagic vesicles and endosomes, as suggested in (**C**) by the LC3-reactive endosomes contacting a DALIS (on the right). (**D**) Abundant endosomal structures and few autophagic vesicles (arrowheads) are also found in the cytoplasm of a DC cell treated for 3 days with GM-CSF plus IFNα. (**E**) A large DALIS in the cytoplasm of another cell, treated as in (**D**), which is enlarged in (E1) to show vesicles and dense bodies embedded in an amorphous component. (**F**) An LPS treated IL4-DC showing PaCS (enlarged in F1) and aggregated endosomes (enlarged in F2). PaCS are negative for K63-linked polyubiquitin chains antibody (F1) while some endosomes (arrowheads) (F2) as well as a DALIS (**G**, from an adjacent cell) show a clear reactivity. (**H**) The enlarged cytoplasm of another cell treated as in (**F**) and (**G**) shows p62 reactivity of an endosome (arrow) and lack of reactivity of an adjacent PaCs. m, mitochondria.

**Figure 7:**
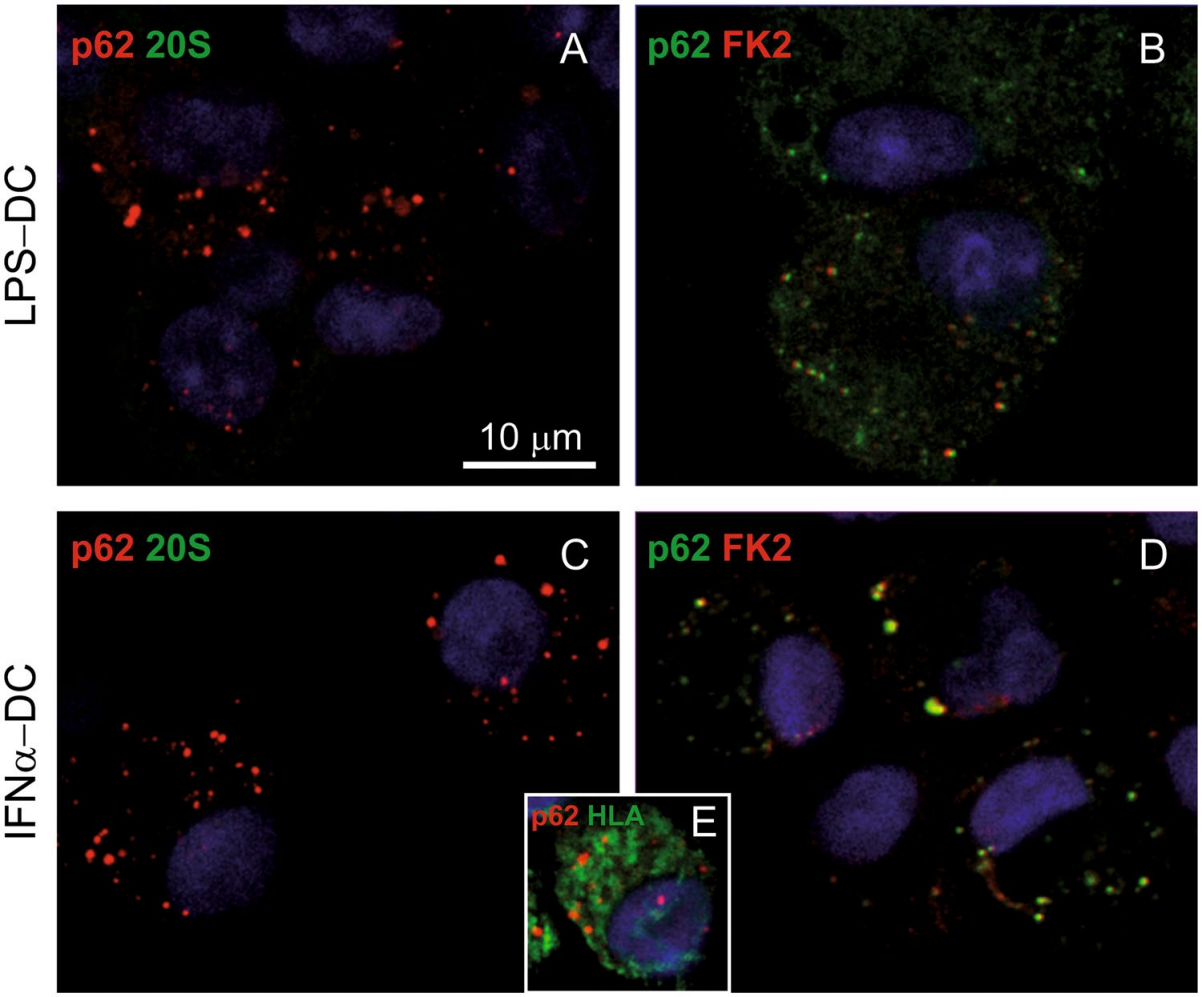
Confocal microscopy of DALIS in GM/LPS-DC and IFN-DC. (**A,B**) Cells treated with GM-CSF for 5 days and then with LPS for 7 h, cytospinned and processed following the common sample preparation for confocal microscopy procedure as in the case of Fig. 3A. Note in (**A**) the p62-immunostained cytoplasmic bodies (red), mostly corresponding to DALIS, and the lack of 20S (green) reactivity. (**B**) Cells show colocalization (yellow) of p62 (green) and FK2 (red) in many DALIS-type cytoplasmic bodies. (**C,D**) Cells incubated for 3 days with GM-CSF plus IFNα showed p62-reactive DALIS and lack of 20S-reactive PaCS (**C**), and colocalization of p62 (green) and FK2 (red) in DALIS (**D**). (**E**) IFN-DC showed partial colocalization (yellow) between p62 (red) and class II HLA (green).

During GM-CSF treatment, we observed DC differentiation that resembled morphologically that obtained with GM-CSF plus IL-4, although it progressed more slowly. After 5 days treatment, TEM showed an increased proportion of endosomal and autophagic structures scattered in the cytoplasm (Fig. 6A). After LPS treatment, such structures coalesced to form large (0.5–4 μm in diameter) vesicular aggregates with interposed amorphous material, reactive for ubiquitin (clone Z0458), K63-linked polyubiquitin chains, p62, and LC3 antibodies, although unreactive for proteasome, and HSP70 antibodies (Fig. 6B,C). These structures closely resembled the vesicular DALIS previously characterized ultrastructurally in LPS-treated murine DC^18^ and rat macrophages^22^. In keeping with the original description of DALIS by Leluard *et al*.^14^ conventional confocal microscopy of GM-CSF differentiated, LPS maturated human DC showed large ubiquitin-reactive (FK2 or Z0458 antibodies) cytoplasmic bodies, also reactive for p62 and LC3, while being unreactive for proteasome and HSP70 antibodies (Fig. 7A,B).

#### GM-CSF plus IFNα

Three days treatment with GM-CSF plus IFNα induced only partial DC differentiation with moderate amounts of cytoplasm and cell processes. Numerous endosomes and autophagosomal vesicles were seen by TEM (Fig. 6D), which in 20–30% of cells sections, coalesced to form characteristic ubiquitin, p62 and LC3-reactive, DALIS-type bodies enclosing vesicular and membrane-delimited structures embedded in an amorphous component (Fig. 6E). Conventional confocal microscopy showed that, like those of GM/LPS DC, DALIS of IFN-DC reacted with p62 (Fig. 7C), LC3, ubiquitin (FK2 or Z0458) (Fig. 7D), and class II HLA (Fig. 7E) antibodies, while failing to react with proteasome antibodies.

### Combined PaCS and DALIS development in LPS-treated IL4-DC

In DC fully differentiated with GM-CSF plus IL4, additional LPS treatment for 7–10 hours was found to preserve PaCS, while producing an increase in cytoplasmic endosomes and autophagosomal vesicles, partly aggregated to form DALIS bodies. This allowed direct comparison of PaCS and DALIS structures and immunoreactivities inside the same cell preparation, including DALIS or endosomes reactivity, and PaCS unreactivity, for K63 polyubiquitin chains and p62 protein (Fig. 6F–H). No PaCS development was observed in cells treated with GM-CSF alone or GM-CSF plus IFNα, with or without LPS treatment and DALIS development.

## Discussion

**Figure 8:**
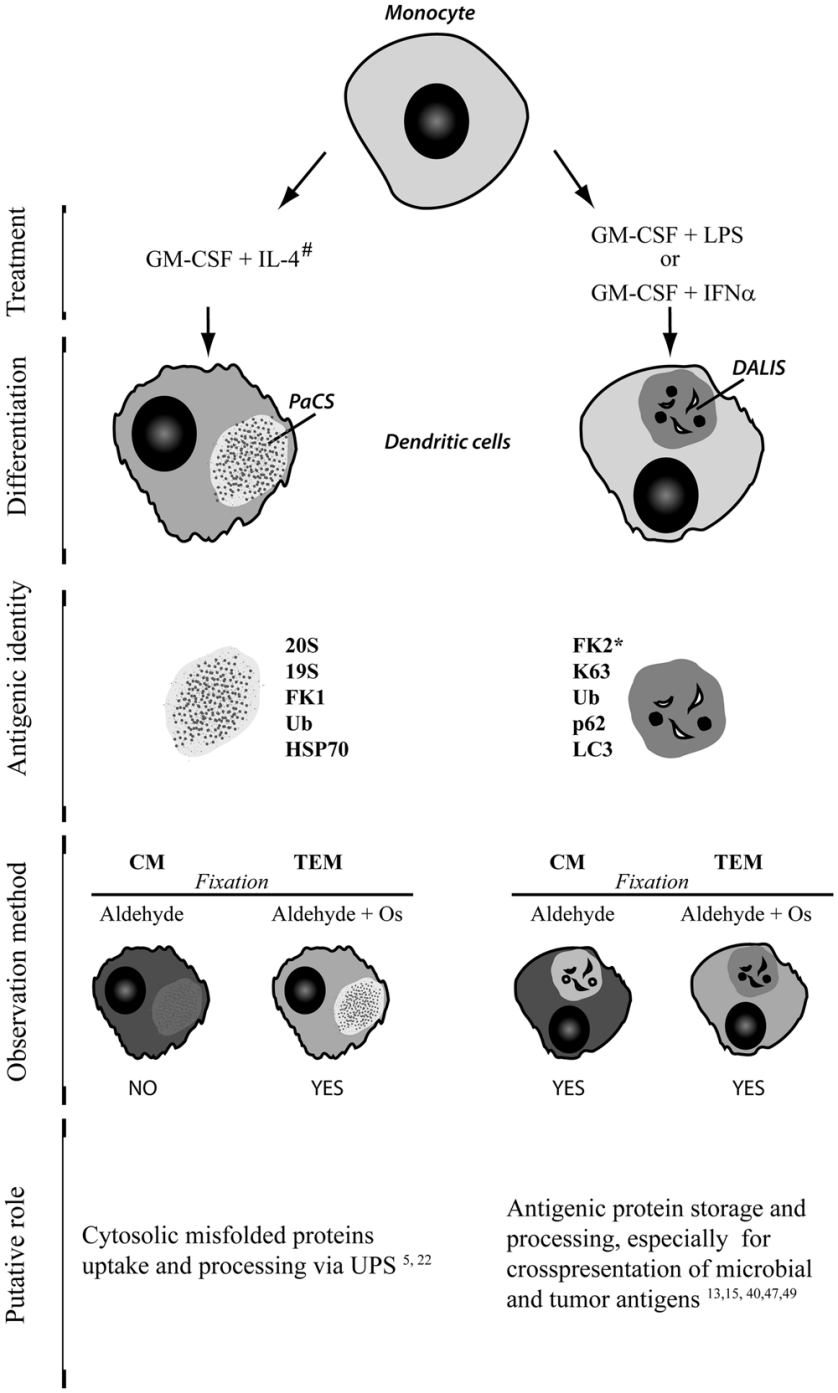
PaCS and DALIS characterization. See text for description. ^#^When LPS is added to differentiated IL4-DC, both PaCS and DALIS can be obtained. *For light microscopy only. CM, confocal microscopy. Os, osmium.

In this study, we show that PaCS and DALIS are distinct DC structures. Indeed, they were found to differ in the following aspects (Fig. 8). (i) Development phase: during differentiation for PaCS and during maturation for DALIS. (ii) Induction treatment: IL4 added to GM-CSF for PaCS, and LPS or IFNα added to GM-CSF for DALIS. (iii) Ultrastructure: PaCS consisted of a uniform collection of barrel-like particles (possibly corresponding to 26S proteasome), whereas DALIS were formed by a heterogeneous accumulation of vesicles with content of variable density and interposed amorphous material. (iv) Cytochemical content: although both PaCS and DALIS contained ubiquitinated proteins, K63-linked polyubiquitin chains were exclusively detected in DALIS, while PaCS only contained proteasomes and HSP70, and DALIS only contained p62 protein and LC3. (v) Solubility and fixability: PaCS contents were highly soluble in ordinary aqueous aldehyde solutions. PaCS preservation in cells and tissues required strong fixation, such as double aldehyde/osmium treatment. DALIS were insoluble in ordinary aqueous fixatives, which allowed their preservation in ordinary light microscopy preparations.

We provide direct evidence that PaCS are induced by cytokines in human DC undergoing differentiation from their monocyte precursors, by combined treatment with GM-CSF plus IL-4. This finding confirms previous suggestions on a possible role of cytokine/trophic factors in PaCS development, based on PaCS detection *in vivo* ^1, 4, 5, 21^ under conditions known to be associated with overexpression/overfunction of cytokines and trophic factors and/or their receptors^7–10^. The present demonstration of PaCS induction by cytokines in cultured DC fits with our *in vivo* detection of PaCS (unpublished data)in H.pylori-infected DC of human gastric mucosa^23^.

Immunogold TEM of PaCS at an early stage of development showed initial accumulation of proteasome-reactive barrel-like particles and polyubiquitinated proteins in close connection with free ribosomes in the absence of ER cisternae. This finding suggests a relationship between PaCS development and cytokine-promoted cytosolic protein neosynthesis. Protein neosynthesis is known to be active during DC differentiation^15, 24^, especially with combined GM-CSF and IL-4 treatment^25, 26^. Evidence of an increase in proteasome biosynthesis (especially immunoproteasomes) has been previously demonstrated in DC obtained after culturing with GM-CSF and IL-4^27, 28^. These findings are confirmed by the present investigation. In addition, a parallel behavior has been found in cytokine-treated cells between proteasomes or polyubiquitinated proteins in cell lysates and the respective levels of PaCS development.

Stimulation of protein synthesis is usually associated with increased production of defective ribosomal products or misfolded proteins, with which Hsp40 and Hsp70 interact, while still linked to or just after release from the ribosomes. This helps to correct protein folding or, in case of failure, to promote protein polyubiquitination and proteasome-dependent degradation^29, 30^. Indeed, accumulation of polyubiquitinated (misfolded) proteins are known to stimulate neosynthesis of proteasome components and promote their assembly into the active 26S proteasome machinery^31, 32^. It has been suggested that the latter is equivalent *in vivo* to the PaCS proteasome-reactive, barrel-like particles^5^. Thus, PaCS could be regarded as an early site of protein quality control taking care of cytosolic proteins. This interpretation of the role of PaCS fits with their occurrence in various types of fetal or pathologic cells^1–4, 6, 21^, under conditions characterized by increased/deranged protein synthesis and increased ubiquitin proteasome system (UPS) content^6, 10, 33–35^.

An interesting finding was the regression, up to complete disappearance, of PaCS after cytokine withdrawal, confirming PaCS cytokine dependence and showing involvement of autophagy and its cargoes destined for late endosomes and lysosomal degradation. This finding links together the two main protein degradation pathways, UPS and autophagy. It may provide a morphologic basis for the recently described functional interactions between the two systems^36^, while fitting with previous *in vivo* observations on PaCS autophagy in chronic myeloid leukemia cells^6^.

In IL4-DC, we found PaCS-filled cytoplasmic membrane blebs apparently detaching from the cell to form membrane vesicles corresponding in size (mostly 100–500 μm in diameter) and shape to exovesicles^37^ or ectosomes^20^. This suggests a role for PaCS-storing blebs in intercellular antigen transport. Indeed, DC blebs and vesicles, filled with polyubiquitinated proteins, proteasomes, and chaperone molecules, recall the proteasome-filled blebs reported by Pitzer and coworkers^38^ in early apoptotic cells, as well as PaCS-filled blebs and vesicles previously shown to detach from neoplastic cells^4, 6^. These structures are known to deliver antigenic materials to APC for presentation to CD8^+^ T cells in a class I MHC background^6, 37, 39–41^. It is generally agreed that proteasome proteolysis is required for class I presentation of endogenous peptides, and cytosolic proteasomes have long been implicated in the process^42–44^. Being a focal concentration of cytosolic proteasomes and polyubiquitinated proteins, which are potential substrates for antigen peptide generation, PaCS could also be involved in this process.

Compared to GM-CSF plus IL-4, the incomplete or delayed DC differentiation seen morphologically and functionally with GM-CSF alone or with IFNα, is not surprising because IL4-induced promotion of DC differentiation is known^12, 45^. It seems unlikely that this change by itself can explain the failure of such DC to develop PaCS, given the early (1–2 days) appearance and rapid, massive expansion of PaCS in IL4-DC, even before they reach full differentiation (5 days). A crucial role of IL-4 itself seems more likely, which remains to be elucidated. It should be added that other interleukins, beside IL-4, have also been shown to induce PaCS in pertinent cell types, such as IL-2 and IL-15 in NK-cells during specific morphologic and functional differentiation from their blood precursors^5^.

The increased content of endosomal structures that we found in LPS treated DC is in keeping with the role taken by these structures in antigen processing^13, 46^. Endosomal vesicles aggregation into DALIS after LPS treatment fits with the proposed role for this body as transient deposit of potentially antigenic polyubiquitinated proteins on their way to processing and presentation^14, 15, 18, 47^. In addition, the selective enrichment in K63-linked polyubiquitin proteins we found in both endosomes and DALIS fits with the ability of K63 chains to direct proteins toward endosomal/lysosomal trafficking by selectively binding the ESCRT (Endosomal Sorting Complex Required for Transport) proteins, which also prevent their interaction with proteasome^48^. Thus, the unreactivity of PaCS for K63 chains outlines its divergent function in respect to endosomes and DALIS, while supporting its link with a proteasome-related degradative function.

A similar process of endosomal vesicles development and aggregation into DALIS was observed in IFN-DC, in the absence of microbial LPS “maturation” treatment. This finding, which was obtained after only 3 days treatment, and despite morphologic and immunophenotypic signs of incomplete DC differentiation, should be coupled with the effectiveness of such cells in mediating cross-presentation of viral and tumor antigens^13, 19^. Indeed, IFN-DC were found to be more effective than IL4-DC in priming cytotoxic anti-leukemia T cells^49^. It seems likely that the IFNα-induced, endosome/DALIS-enriched DC differentiation and maturation contribute to this special kind of immune function, where delayed intracellular protein degradation results in relatively long-lasting antigen presentation^13, 15, 40, 47^. It should be stated that, despite their lower expression of classic DC morphology and CD1a marker, compared to IL4-DC, IFN-DC showed comparable expression of membrane molecules directly involved in antigen presentation, such as CD80/CD86 and HLA-DR. More generally, it seems that the consistent morphologic and cytochemical differences, including DALIS versus PaCS development, we observed among DC subsets, may find pertinent functional counterparts in their recently reported metabolic and immunologic differences^13, 25, 26, 50^.

In conclusion, PaCS is a distinctive cytoplasmic structure that, in human DC during differentiation from monocyte precursors, is selectively induced by a combination of GM-CSF and IL-4, while regressing after cytokine withdrawal. PaCS appear in close topographic connection with free polyribosomes, and contain chaperone molecules, polyubiquitinated proteins and proteasome-reactive, barrel-like particles. This suggests a direct role in handling non-native proteins, when newly formed in excess during specific cytokine/trophic factor stimulation *in vitro*, or in neoplastic and non-neoplastic cells under chronic pathologic conditions *in vivo*. DALIS differ morphologically, cytochemically and functionally from PaCS. DALIS originate in DC when, after differentiation with GM-CSF, they are matured with microbial products, or when differentiation and maturation of their antigen processing and presenting activity are obtained simultaneously via GM-CSF and IFNα exposure. Although our findings suggest distinct functions for DALIS and PaCS, further experimental work and mechanistic investigation are required to clarify their specific role in the different types of DC-induced immune response.

## Materials and Methods

### Human DC generation

DC were generated from purified blood monocytes derived from buffy coats of healthy donors. Buffy coat units were released by Blood Bank “Centro di Lavorazione e Validazione “, Transfusion Service, Policlinico San Matteo (Pavia, Italy), according to Article 8 of the Decree 2 november 2015 of Italian Department of Health, with blood donor specific informed consent (including the use for research) implemented by Blood Bank Conference of Regione Lombardia. This study is part of a line of research dealing with PaCS and DALIS investigation that has been approved by the Ethics Committee of Fondazione IRCCS Policlinico San Matteo (Pavia, Italy). Purified monocytes were obtained with anti-CD14 magnetically labeled microbeads (Miltenyi Biotec, Germany) and cultured in RPMI supplemented with 10% FCS and 2 mM glutamine (complete medium), using different culture conditions: (A) IL-4+GM-CSF (IL4-DC); (B) GM-CSF alone (GM-DC); and (C) IFNα+GM-CSF (IFN-DC). In all culture conditions, GM-CSF (CellGenix GmbH, Freiburg, Germany) was added at a concentration of 800 U/ml, while IL-4 and IFN-α (Miltenyi Biotec) were used at 500 U/ml and 10^4^ U/ml, respectively. IL4-DC were evaluated at various times after differentiation, starting from 7-h to 6-day culture. IFN-DC were evaluated after 3-day culture, which was the optimal incubation time for obtaining functionally active DC while avoiding apoptosis^19^. Surface antigens expressed by DC were evaluated by flow cytometry (Navios flow cytometer equipped with Kaluza 1.1 software; Beckman Coulter, Brea, CA, USA) using a specific monoclonal antibody, as described previously^51^. Cytofluorimetric analysis was performed gating all marked cells and evaluating the expression of every surface antigen. Phenotypic analysis included FITC- and PE-labeled anti-CD1, anti-CD14, anti-CD80, anti-CD86 and anti-HLA-DR (BD Pharmingen, San Diego, CA, USA). To evaluate the role of cytokine withdrawal, after 5-day culture, IL4-DC were recovered, washed and plated in complete medium alone, without addition of cytokines. IL4-DC were then evaluated at different times from 1-day to 5-day culture. To evaluate LPS-induced maturation, DC after 5-day culture were recovered and incubated for an additional 7–10 h in complete medium supplemented with LPS (100 ng/ml).

### Antibodies

The following primary antibodies were used for confocal microscopy immunofluorescence, TEM immunocytochemistry and protein lysate immunoblotting: mouse monoclonal anti-polyubiquitinated proteins (FK1 clone), mouse monoclonal anti-mono- and polyubiquitinated proteins (FK2 clone), rabbit polyclonal anti-20S proteasome core subunits, mouse monoclonal anti-proteasome β5i subunit, mouse monoclonal anti-K63-linked polyubiquitin chains (clone HWA4C4) and mouse monoclonal anti-HSP90 (all from Enzo Life Sciences International, Plymouth Meeting, PA, USA); rabbit polyclonal anti-20S proteasome core subunits, rabbit polyclonal and mouse monoclonal anti-p62 and goat and rabbit polyclonal anti-HSP70 (Santa Cruz Biotechnology, Santa Cruz, CA, USA); rabbit polyclonal anti-ubiquitin (clone Z0458) and mouse monoclonal anti-human class II HLA-DP, -DQ and -DR antigens (clone CR3/43) (Dako, Glostrup, Denmark); mouse monoclonal anti-GAPDH (Abcam, Cambridge, UK); rabbit polyclonal anti-19S proteasome S2-subunit (Calbiochem, Merck–Millipore, Darmstadt, Germany); rabbit polyclonal anti-HSP40 (LS Bio, Seattle, WA, USA); rabbit polyclonal anti-LC3 (Novus Biological, Cambridge, UK). As secondary antibodies for confocal microscopy we used Alexa-488-labeled anti-mouse IgG or anti-rabbit IgG (Life Technologies, Paisley, UK), aminomethylcoumarin-acetate-labeled anti-mouse IgG/IgM, DyLight-488-labeled anti-mouse IgM, Texas-Red-labeled anti-mouse IgG, and Cy5-labeled anti-rabbit IgG (all from Jackson Immunoresearch, West Grove, PA, USA). For ultrastructural immunocytochemistry, anti-rabbit or anti-mouse IgG or IgM secondary antibodies labeled with colloidal gold particles (5–20 nm diameter) (British BioCell, Cardiff, UK; and Aurion, Wageningen, Netherlands) were used.

### TEM and immunocytochemistry

For TEM, the cells were pelleted and fixed for 3–4 h at 4 °C with 2.5% glutaraldehyde and 2% formaldehyde in 0.2 M cacodylate buffer (pH 7.3), followed by 1.5% osmium tetroxide for 1 h at room temperature. Alternatively, they were fixed for 1 h at 4 °C in a freshly prepared mixture of one part 2.5% glutaraldehyde and two parts 1% osmium tetroxide in cacodylate buffer. After dehydration in ethanol and propylene oxide, the specimens were embedded in Epon–Araldite resin. Semithin (~1 μm) resin sections were stained with toluidine blue in a pH 8.0 borax solution or processed for confocal immunofluorescence microscopy, as described below. Consecutive thin (~70 nm) sections were stained with uranyl acetate–lead citrate or underwent immunogold procedures followed by uranyl acetate–lead citrate staining^1^. Specimens were analyzed by a Jeol JEM-1200 EX II transmission electron microscope equipped with an Olympus CCD camera (Mega View III). The aldehyde–osmium fixation, often required for unequivocal interpretation of TEM immunogold findings, blocks the reactivity of some antibodies (e.g. FK2 antibody); however, full reactivity is retained by the same antibodies for conventional confocal microscopy of aldehyde-fixed cells^5^. Thus, for immunodetection of ubiquitinated proteins in aldehyde–osmium resin sections, we relied on FK1 and Z0458 antibodies, while in formaldehyde-fixed sections for conventional confocal microscopy immunofluorescence, we also used FK2 antibody, which is a preferred marker of DALIS^14^.

Quantitative analysis of PaCS development in different cell populations was performed on 100 randomly selected cell profiles by ImageJ software and expressed as percentage of PaCS area of the total cytoplasmic area.

### Confocal microscopy

DC were spun and collected on glass slides (cytospin), then immediately fixed with 4% paraformaldehyde for 15 min at room temperature. After washing in PBS, the cells were treated with 50 mM NH_4_Cl in PBS for 5 min to quench free aldehyde groups and permeabilized with PBS containing 0.5% BSA and 0.5% saponin for 5 min (common procedure for confocal microscopy sample preparation). Samples were then incubated for 1 h at room temperature, first with primary antibody and then with fluorescent secondary antibody, as previously described^5^. When necessary, Hoechst 33258 was used for nuclear counterstaining. Semithin sections of DC, fixed and processed as for TEM and embedded in Epon–Araldite resin, were incubated with primary and secondary antibody as described above.

A TCS SP5II confocal laser scanning microscope equipped with PL APO 40 × /1.25 NA and 63 × /1.40 NA oil-immersion objectives (Leica, Heidelberg, Germany) was used. After acquisition, ImageJ software and related plugins (National Institutes of Health) were used for image processing and co-localization.

### Immunoblotting

For protein analysis, DC were washed with PBS containing sodium vanadate and diisopropylfluorophosphate, then lysate on ice in 50 mM HEPES (pH 7.5), 1% Triton X-100, 5 mM EGTA, 50 mM NaCl, 50 mM NaF, 20 mM sodium pyrophosphate, 1 mM sodium vanadate, 2 mM PMSF, 0.2 mg/ml aprotinin/leupeptin, and 8 mM diisopropylfluorophosphate. Lysate was cleared at 10,000 *g* for 15 min and dissociated in SDS sample buffer, then heated at 95 °C for 5 min. Fifty micrograms of protein was separated on a 4–20% precast SDS/polyacrylamide gel (Biorad, Hercules, CA, USA), and transferred to PVDF blotting membranes (GE Healthcare, Little Chalfont, UK). Membranes were blocked with 5% BSA or non-fat milk and incubated with primary antibody (FK1, 1:2000; 20S, 1:2000; HSP70, 1:300; GAPDH, 1:10.000) overnight at 4 °C. After thorough washing, membranes were incubated with the appropriate anti-rabbit, anti-mouse, or anti-goat peroxidase-conjugated secondary antibodies (Millipore, Billerica, MA, USA; Abcam). Reactive proteins were visualized by chemiluminescent reaction (GE Healthcare).
